# Screening for severe drug-drug interactions in patients with multiple sclerosis: A comparison of three drug interaction databases

**DOI:** 10.3389/fphar.2022.946351

**Published:** 2022-08-05

**Authors:** Michael Hecker, Niklas Frahm, Paula Bachmann, Jane Louisa Debus, Marie-Celine Haker, Pegah Mashhadiakbar, Silvan Elias Langhorst, Julia Baldt, Barbara Streckenbach, Felicita Heidler, Uwe Klaus Zettl

**Affiliations:** ^1^ Division of Neuroimmunology, Department of Neurology, Rostock University Medical Center, Rostock, Germany; ^2^ Ecumenic Hainich Hospital gGmbH, Mühlhausen, Germany

**Keywords:** multiple sclerosis, potential drug-drug interactions, drug interaction databases, medication review, therapy management, patient safety

## Abstract

**Background:** Patients with multiple sclerosis (MS) often undergo complex treatment regimens, resulting in an increased risk of polypharmacy and potential drug-drug interactions (pDDIs). Drug interaction databases are useful for identifying pDDIs to support safer medication use.

**Objective:** To compare three different screening tools regarding the detection and classification of pDDIs in a cohort of MS patients. Furthermore, we aimed at ascertaining sociodemographic and clinical factors that are associated with the occurrence of severe pDDIs.

**Methods:** The databases Stockley’s, Drugs.com and MediQ were used to identify pDDIs by screening the medication schedules of 627 patients. We determined the overlap of the identified pDDIs and the level of agreement in pDDI severity ratings between the three databases. Logistic regression analyses were conducted to determine patient risk factors of having a severe pDDI.

**Results:** The most different pDDIs were identified using MediQ (*n* = 1,161), followed by Drugs.com (*n* = 923) and Stockley’s (*n* = 706). The proportion of pDDIs classified as severe was much higher for Stockley’s (37.4%) than for Drugs.com (14.4%) and MediQ (0.9%). Overall, 1,684 different pDDIs were identified by at least one database, of which 318 pDDIs (18.9%) were detected with all three databases. Only 55 pDDIs (3.3%) have been reported with the same severity level across all databases. A total of 336 pDDIs were classified as severe (271 pDDIs by one database, 59 by two databases and 6 by three databases). Stockley’s and Drugs.com revealed 47 and 23 severe pDDIs, respectively, that were not included in the other databases. At least one severe pDDI was found for 35.2% of the patients. The most common severe pDDI was the combination of acetylsalicylic acid with enoxaparin, and citalopram was the drug most frequently involved in different severe pDDIs. The strongest predictors of having a severe pDDI were a greater number of drugs taken, an older age, living alone, a higher number of comorbidities and a lower educational level.

**Conclusions:** The information on pDDIs are heterogeneous between the databases examined. More than one resource should be used in clinical practice to evaluate pDDIs. Regular medication reviews and exchange of information between treating physicians can help avoid severe pDDIs.

## Introduction

Multiple sclerosis (MS) is a chronic autoimmune disease and the most common cause of non-traumatic neurologic disability in young adults (Filippi et al., 2018). A total of 2.8 million people are estimated to live with MS worldwide (Walton et al., 2020). Inflammation with demyelination, astroglial proliferation (reactive gliosis) and neurodegeneration with axonal and synaptic loss are the pathological hallmarks of the disease (Filippi et al., 2018). The course of MS is different in each patient and can be classified into relapsing-remitting MS (RRMS), primary progressive MS (PPMS) and secondary progressive MS (SPMS) (Lublin et al., 2014). The spectrum of MS phenotypes further includes the clinically isolated syndrome (CIS) (Lublin et al., 2014). The clinical manifestations are very heterogeneous (Zettl et al., 2012). Common consequences of MS include impaired mobility, ataxia/tremor, cognitive dysfunction and pain (Larocca, 2011; Rommer et al., 2019a). The symptoms of MS are frustrating for many patients as they severely limit the quality of their daily lives. One therapeutic approach is offered by the use of disease-modifying drugs (DMDs). DMDs can prevent the development of new lesions in the brain and spinal cord, reduce the frequency of relapses and delay the progression of disability (Rommer et al., 2019b; Hauser and Cree, 2020; Rommer and Zettl, 2022). Additionally, patients with MS often take medications to treat specific disease symptoms (Dargahi et al., 2017), medications for comorbidities as well as complementary and alternative medicines (CAMs) such as vitamin and mineral supplements (Apel-Neu and Zettl, 2008; Kochs et al., 2014; Rommer et al., 2018).

As the world population is getting older on average (Aburto et al., 2020), multimorbidity and consequently polypharmacy are increasingly posing health risks (Payne, 2016; Molokhia and Majeed, 2017). Therefore, interest in potential drug-drug interactions (pDDIs) is rising among physicians, and an appropriate management of medications that may interact is becoming more and more relevant. pDDIs can generally be divided into two different classes: pharmacokinetic and pharmacodynamic interactions. Pharmacokinetic pDDIs affect the liberation, absorption, distribution, metabolism and elimination of drugs, e.g., through the inhibition or induction of metabolic enzymes like the cytochrome P450 (CYP) isozymes or through reduced absorption due to complexation of active substances (Koziolek et al., 2019; Bechtold and Clarke, 2021). Pharmacodynamic pDDIs refer to the influence on the mode of action of drugs, e.g., through additive effect enhancement or antagonistic effect reduction (Niu et al., 2019). In the case of an improper therapy management, there is a risk of overdosed or underdosed therapy, and side effects may occur due to pDDIs.

There are numerous online tools for healthcare professionals and patients to check for pDDIs (Adam and Vang, 2015; Roblek et al., 2015; Kheshti et al., 2016; Hammar et al., 2021). By using these so-called clinical decision support softwares (CDSS) and drug-drug interaction databases (DDIDs), the risk assessment of combined pharmacotherapy is facilitated. This holds greater safety for patients as dangerous pDDIs can be detected and prevented. However, as several pDDI resources have been developed, the question arises which one to use. Physicians and pharmacists should be aware of the differences between pDDI screening tools and know their advantages and limitations. Previous studies have shown relatively low agreement on the classification of pDDIs among different tools, with the overlap being as low as 5% (Amkreutz et al., 2017; Fung et al., 2017; Prely et al., 2022). It is thus often recommended to use more than one database to increase sensitivity (Smithburger et al., 2010; Wang et al., 2010; Kheshti et al., 2016; Monteith and Glenn, 2019; Sancar et al., 2019; Suriyapakorn et al., 2019; Monteith et al., 2020). It should be also noted that DDIDs often label pDDIs with a higher severity rating than bedside clinicians (Armahizer et al., 2013; Roblek et al., 2015).

The occurrence of pDDIs is a highly relevant issue that has been well studied in certain diseases, such as metabolic syndrome (Suriyapakorn et al., 2019), bipolar disorder (Monteith et al., 2020) and acquired immunodeficiency syndrome (Ramos et al., 2015). However, with respect to MS, the number of studies on pDDIs is low. We have previously examined pDDIs in female MS patients of childbearing age, with a special focus on interactions that might endanger pregnancy (Frahm et al., 2020a). Moreover, we analyzed the contribution of over-the-counter (OTC) drugs to pDDIs (Bachmann et al., 2022), and we compared the risk of pDDIs between MS patients with and without polypharmacy (Debus et al., 2022). In these studies, either one or two DDIDs were used. To our knowledge, there are so far no other studies on pDDIs in unselected patients with MS.

As there might be disease-specific differences in the performance of pDDI screening tools, we here combined the data from our previous works (Bachmann et al., 2022; Debus et al., 2022) to compare the three databases Stockley’s, Drugs.com and MediQ with regard to the identification of pDDIs in MS patients. We further examined the concordance in pDDI severity ratings between the databases. Moreover, we identified the most frequent severe pDDIs in our patients and determined sociodemographic and clinical predictors of having a severe pDDI.

## Materials and methods

### Study population

The patient survey as part of this study was conducted between March 2017 and May 2020 at the Department of Neurology of the Rostock University Medical Center (Germany) and at the Department of Neurology of the Ecumenic Hainich Hospital Mühlhausen (Germany). The patients had to have a diagnosis of a CIS or MS according to the revised McDonald criteria (Thompson et al., 2018). We included data from adult male and female patients, whereas data from minors under the age of 18 were not included. At both centers, the patients were treated as outpatients or inpatients, depending on the individual disease activity and disease progression. Further information on the design of this cross-sectional study are given elsewhere (Bachmann et al., 2022; Debus et al., 2022).

The patients were interviewed while waiting for outpatient appointments and during inpatient stays due to acute disease exacerbation or changes in therapy. Written informed consent was obtained from all patients who agreed to participate in advance. The ethics committees of the University of Rostock and of the State Medical Association of Thuringia approved this study (approval numbers A 2014-0089 and A 2019-0048). We conducted this study in accordance with the current Declaration of Helsinki.

### Data collection

Sociodemographic data (sex, age, years of schooling, educational level, employment status, partnership status, place of residence, number of children and number of siblings), pharmacological data (medications taken with active ingredient, trade name, route of administration and dosage) and clinical data [comorbidities, course of MS, disease duration and disability level according to Kurtzke’s Expanded Disability Status Scale (EDSS)] were obtained using patient records, clinical examinations and structured interviews. The EDSS is the standard instrument for assessing the impairments that can result from MS through neurological examination (Kurtzke, 1983; Kappos et al., 2015). Comorbidity was defined as any additional disease that developed before or during the course of MS and that is not an obvious complication of MS (Magyari and Sorensen, 2020).

From the medication schedules, we captured both on-demand drugs, which are taken irregularly as needed, and long-term drugs, which are taken periodically. More specifically, methylprednisolone was documented as “on-demand drug” when used to treat an acute relapse (Repovic, 2019) and as “long-term drug” when used as repeated pulse therapy for progressive courses of MS (Winkelmann et al., 2016). In addition to recording the use of prescription drugs (Rx), we also explicitly asked the patients about their use of non-prescription drugs (OTC) as well as CAMs like herbal medicines or dietary supplements (Evans et al., 2018; Rommer et al., 2018). Note that some drugs are available as both Rx and OTC preparations, depending on the dosage (e.g., ibuprofen). All drugs were recorded independently of the treatment goals and thus included DMDs for MS, medications to treat disease symptoms as well as medications for comorbidities.

### Assessment of potential drug-drug interactions

For the comprehensive analysis of pDDIs, every patient’s medication plan was screened using three different DDIDs: Stockley’s, Drugs.com and MediQ. Stockley’s Interactions Checker is an English-language subscription-based online pDDI tool with over 85,000 deposited interactions. It is published by the Royal Pharmaceutical Society and updated monthly. The pDDI severity levels are divided into three categories: mild (minimal clinical relevance), moderate (moderate clinical relevance) and severe (high clinical relevance) interactions. Furthermore, Stockley’s provides information about potential drug-food/beverage/smoking and drug-herb interactions. This tool is based on “Stockley’s Drug Interactions”, the most comprehensive international reference book on drug interactions (Preston, 2020), and primarily aimed at healthcare professionals.

Drugs.com Drug Interactions Checker, edited by the Drugsite Trust, is a free English-language website with information on ∼24,000 drugs and herbal medicines. This database classifies pDDIs into three severity levels: minor (minimally clinically significant), moderate (moderately clinically significant) and major (highly clinically significant). The database is aimed at both consumers and medical professionals as explanations of pDDIs are available according to prior medical knowledge. Drugs.com also displays information on potential drug-food/alcohol interactions. A country-restricted mobile app is available. The free accessibility and patient orientation of this DDID clearly sets it apart from other pDDI screening tools.

MediQ is a Swiss web-based tool containing more than 2,000 active substances and more than 50,000 interactions, including not only pDDIs but also drug-food, drug-beverage, and drug-polymorphism interactions (Suter et al., 2013). The latter allow to evaluate the pharmacogenetic effects of patient-specific genetic factors. MediQ is designed for medical staff and is only accessible with a subscription. It is only available in the German language. The pDDI severities are rated as low danger, average danger and high danger of interaction. Furthermore, MediQ distinguishes whether a pDDI is currently ruled out (i.e., there is no known interaction) or whether a drug combination has not yet been assessed by the MediQ operators (i.e., there is no data in the database). Users can request combinations of drugs to be included in the database. MediQ is one of the most commonly used German-language tools for identifying pDDIs. In a study comparing five German-language tools, MediQ was the one with the most complete results (Hahn and Roll, 2018).

The screening for pDDIs was conducted from May 2020 to November 2020 by entering the trade name of each drug in the search field of each database. If the trade name was not found, we entered the generic name(s) of the active ingredient(s) contained in the respective drug. The route of administration (e.g., oral or dermal) was entered as well if possible. pDDIs that were detected in the DDIDs were subsequently recorded in Excel spreadsheets and sorted by severity. To facilitate the interpretation of the database comparisons, we decided to consistently refer to the three pDDI severity levels as mild, moderate and severe as they are called in Stockley’s, instead of using different labels (such as minor/low) per database. With regard to MediQ, we considered the category “no data available” as equivalent to the category “no known interaction” for simplicity.

### Data analysis

The data were prepared with IBM SPSS Statistics version 27, Microsoft Excel 2010 and ONLYOFFICE 7.0. Descriptive statistics and further data analyses were performed in R version 3.6.0. We first determined the number of different pDDIs (i.e., without repetitions if they occurred in more than one patient) found with Stockley’s, Drugs.com and MediQ. The relative proportions of mild, moderate and severe pDDIs per database were then visualized using doughnut plots. The overlap of pDDIs from the 3 databases was analyzed with the R package VennDiagram (Chen and Boutros, 2011). Concordance rates were calculated by dividing the number of identical pDDI severity ratings by the number of pDDIs that were detected in each of two databases being compared. Cohen’s kappa coefficients (κ) were also computed to summarize the agreement among the databases. The severe pDDIs were drawn as a network using Cytoscape 3.9.0 (Shannon et al., 2003) with yFiles layout algorithms. Binary logistic regression analyses were performed to predict the patients’ risk of having a severe pDDI. The numerical, ordinal and dichotomous variables were included either separately (univariable models) or jointly (multivariable model). The latter was performed by bidirectional stepwise model selection based on the Akaike information criterion (AIC) (Akaike and Lovric, 2011) using the R package MASS. The resulting odds ratios (ORs) were visualized as forest plots with the R packages sjPlot and ggplot2 (Wickham, 2016). The corresponding statistical tests were exploratory in nature, and therefore the significance level was set at *α* = 0.05. We checked for collinearities in the data by calculating the variance inflation factor (VIF) for each independent variable with the mctest R package. Scatterplots were used to display the relationship between age and number of drugs taken with pDDI count. Exponential curves were fitted to the data, and 95% confidence intervals of the fitted curves were calculated by performing bootstrap resampling.

## Results

### Patient cohort

A total of 627 patients were included in this study (Table 1). The patient cohort was composed of cases with CIS (*n* = 27), RRMS (*n* = 388), SPMS (*n* = 154), and PPMS (*n* = 58). The proportion of women was 70.3% (*n* = 441). The age of the patients ranged from 19 to 86 years (mean ± standard deviation: 48.6 ± 13.3). There were 465 patients (74.2%) who lived in a partnership and 162 patients (25.8%) who lived alone. A large proportion of the subjects resided in a rural area (*n* = 224), whereas the others lived in a provincial town (*n* = 108), medium-sized town (*n* = 112) or city (*n* = 183). With regard to the level of education, the patients had either no training (*n* = 19), a qualification as a skilled worker (*n* = 398) or a degree from a technical college (*n* = 89) or university (*n* = 121). The average EDSS score of the patients was 3.6 ± 2.1 (range: 0–9) at a median disease duration of 10 years (range: 0–52). Most of the patients (*n* = 443, 70.7%) had comorbidities in addition to MS. Only seven and 52 patients received no or only one drug, respectively, whereas most patients (*n* = 568, 90.6%) took at least two drugs and thus were at risk of pDDIs. The average number of drugs taken per patient was 5.3 ± 3.3 (range: 0–19). For further details on the clinical, demographic and medication data of the patients, the reader is referred to our previous publications (Bachmann et al., 2022; Debus et al., 2022).

**TABLE 1:** Sociodemographic, clinical and medication data of the patient cohort (*N* = 627).

Parameter	*N* (%) or range	Mean (SD) or median
Sex		
Female	441 (70.3%)	
Male	186 (29.7%)	
Age [in years]	19–86	48.6 (13.3)
School years	6–18	10.5 (1.3)
Educational level		
No training	19 (3.0%)	
Skilled worker	398 (63.5%)	
Technical college	89 (14.2%)	
University	121 (19.3%)	
Employment status		
In training	7 (1.1%)	
In studies	6 (1.0%)	
Employed	269 (42.9%)	
Unemployed	25 (4.0%)	
Retired	304 (48.5%)	
Others	16 (2.6%)	
Partnership		
No	162 (25.8%)	
Yes	465 (74.2%)	
Place of residence		
Rural area	224 (35.7%)	
Provincial town	108 (17.2%)	
Medium-sized town	112 (17.9%)	
City	183 (29.2%)	
Number of children	0–4	1
0	169 (27.0%)	
1	170 (27.1%)	
≥2	288 (45.9%)	
Number of siblings	0–13	1
0	71 (11.3%)	
1	305 (48.6%)	
≥ 2	251 (40.0%)	
EDSS score [points]	0–9.0	3.5
Disease duration [in years]	0–52	10
Disease course		
CIS	27 (4.3%)	
RRMS	388 (61.9%)	
SPMS	154 (24.6%)	
PPMS	58 (9.3%)	
Comorbidities	0–9	1
No	184 (29.3%)	
Yes	443 (70.7%)	
Number of drugs taken	0–19	5
0	7 (1.1%)	
1–4	286 (45.6%)	
5–9	261 (41.6%)	
≥ 10	73 (11.6%)	

CIS, clinically isolated syndrome; EDSS, Expanded Disability Status Scale; PPMS, primary progressive multiple sclerosis; RRMS, relapsing-remitting multiple sclerosis; SD, standard deviation; SPMS, secondary progressive multiple sclerosis.

### Comparison of potential drug-drug interactions information from different sources

**FIGURE 1:**
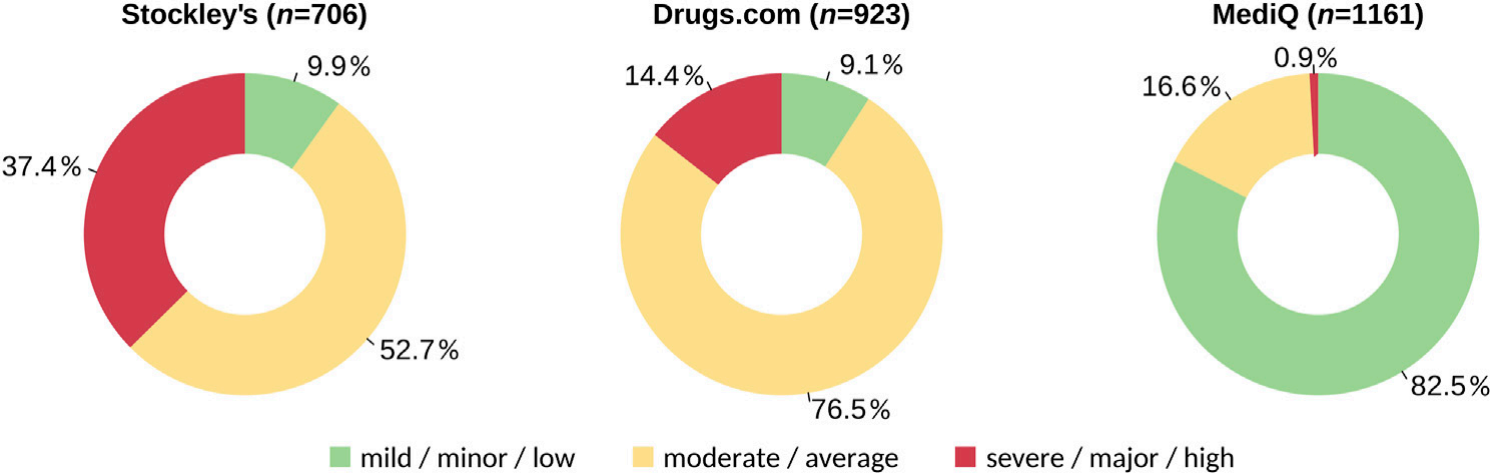
Proportions of mild, moderate and severe potential drug-drug interactions by database. The medication schedules of 627 patients with CIS/MS were evaluated with respect to pDDIs using three databases. The doughnut plots show the proportions across all different pDDIs found. CIS, clinically isolated syndrome; MS, multiple sclerosis; pDDI, potential drug-drug interaction.

**FIGURE 2:**
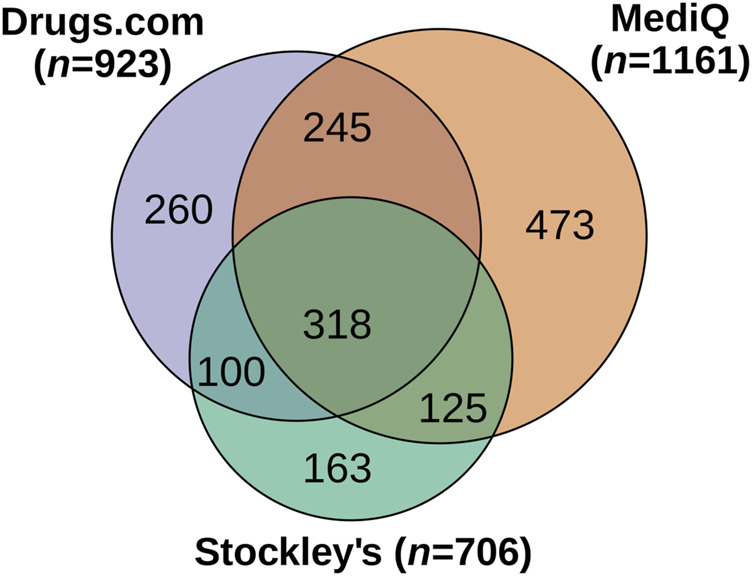
Overlap of potential drug-drug interactions among the three databases. A total of 1,684 different pDDIs were identified for the patient cohort (*N* = 627). The Venn diagram shows the intersections of pDDIs between the databases. The circles and intersecting sets are drawn approximately to scale. pDDI, potential drug-drug interaction.

**FIGURE 3:**
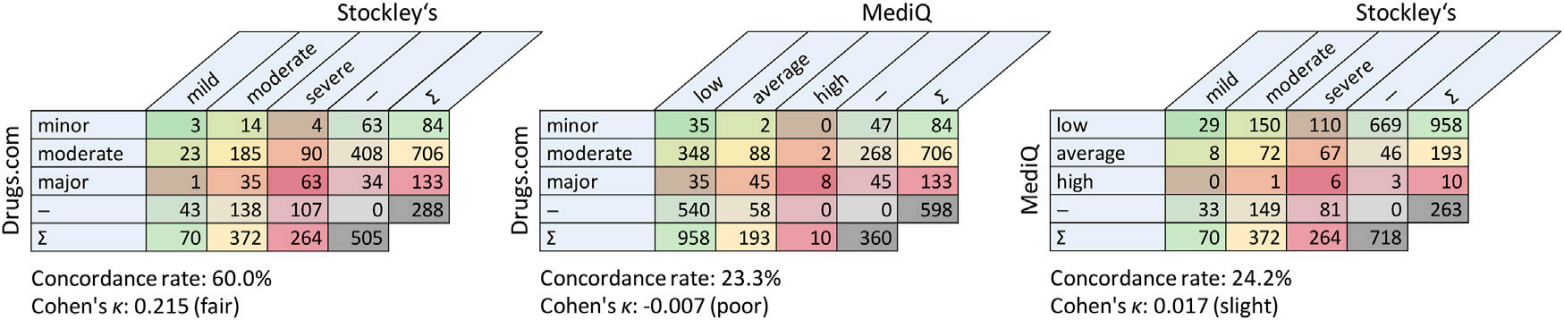
Pairwise comparisons of the databases in terms of reported potential drug-drug interactions by degree of severity. The crosstabs show the overlaps related to different pDDIs. The cells in the tables are color-coded according to pDDI severity from mild (green) to severe (red). — = not recorded in one of the two databases; ∑ = sum per row/column; pDDI, potential drug-drug interaction.

The database-driven screening revealed pDDIs for 280 different drugs. We found 706 different pDDIs with Stockley’s, 923 different pDDIs with Drugs.com and 1,161 different pDDIs with MediQ However, while fewer pDDIs were found using Stockley’s, 264 of the pDDIs from this database (37.4%) were rated as severe. In comparison, only 10 of the pDDIs from MediQ (0.9%) were classified as severe (Figure 1). In total, 1,684 different pDDIs were identified with the three drug interaction databases. The consistency in detecting pDDIs was relatively low: Only 318 pDDIs (18.9%) were reported in all databases, and each database specified pDDIs that were not contained in the other two databases. The largest overlap was noticed for Drugs.com vs. MediQ (563 different pDDIs) (Figure 2). With regard to the pDDI severity ratings, there was a greater agreement for Drugs.com vs. Stockley’s. For these, the severity ratings concordance rate was 60.0%. The respective rate was lower for Drugs.com vs. MediQ (23.3%) and MediQ vs. Stockley’s (24.2%) because the pDDIs were typically reported with a lower severity in MediQ (Figure 3). As many as 110 different pDDIs that were classified as mild in MediQ were severe according to Stockley’s. On the other hand, there were three severe pDDIs from MediQ that were not detected with Stockley’s (amantadine < = > amitriptyline, cannabidiol < = > sertraline and citalopram < = > tamoxifen). Only 55 of the 1,684 different pDDIs (3.3%) have been reported with the same severity level across all databases (i.e., 17.3% of the 318 common pDDIs).

### Severe potential drug-drug interactions in patients with multiple sclerosis

The number of different severe pDDIs was 264 for Stockley’s, 133 for Drugs.com and 10 for MediQ. Overall, 336 different pDDIs were severe according to at least one of the databases (Supplementary Figure S1). A subset of 271 pDDIs were classified as severe in only one database, 59 pDDIs were classified as severe in two databases and six pDDIs were consistently classified as severe in all three databases (citalopram with ciprofloxacin, doxepin, flecainide, levofloxacin, ondansetron and quetiapine). Citalopram was involved in 33 different severe pDDIs. Ibuprofen and methylprednisolone were also frequently involved in pDDIs, with 23 and 22 severe pDDIs, respectively. Forty-three severe pDDIs occurred in three or more of the 627 patients (Table 2). The most common severe pDDI was acetylsalicylic acid < = > enoxaparin, which was recorded for 21 patients. Stockley’s and Drugs.com yielded 47 and 23 severe pDDIs, respectively, that were not included in the other databases (Table 3). Among the drugs that were associated with severe pDDIs, there were also several DMDs for the therapy of MS: cladribine, fingolimod, interferon beta, mitoxantrone, natalizumab and teriflunomide.

**TABLE 2:** Severe potential drug-drug interactions that were found for at least 3 patients.

Drug-drug interaction	Stockley’s	Drugs.com	MediQ	Frequency, *n* (%)
Acetylsalicylic acid < = > Enoxaparin	moderate	severe	moderate	21 (3.3%)
Enoxaparin < = > Ibuprofen	moderate	severe	moderate	16 (2.6%)
Baclofen < = > Ibuprofen	severe	—	mild	15 (2.4%)
Ibuprofen < = > Methylprednisolone	severe	moderate	—	14 (2.2%)
Enoxaparin < = > Ramipril	severe	moderate	mild	13 (2.1%)
Citalopram < = > Methylprednisolone	severe	—	moderate	10 (1.6%)
Dipyrone/metamizole < = > Methylprednisolone	severe	—	mild	9 (1.4%)
Methylprednisolone < = > Solifenacin	severe	—	—	9 (1.4%)
Acetaminophen/paracetamol < = > Ibuprofen	severe	—	mild	7 (1.1%)
Citalopram < = > Fingolimod	severe	severe	moderate	7 (1.1%)
Interferon beta-1a < = > Ramipril	severe	moderate	—	7 (1.1%)
Mitoxantrone < = > Ondansetron	severe	—	—	7 (1.1%)
Acetylsalicylic acid < = > Ibuprofen	severe	severe	moderate	6 (1.0%)
Amlodipine < = > Simvastatin	mild	severe	moderate	6 (1.0%)
Citalopram < = > Ibuprofen	severe	moderate	mild	6 (1.0%)
Ibuprofen < = > Teriflunomide	—	severe	—	6 (1.0%)
Acetylsalicylic aci < = > Dipyrone/metamizole	severe	—	moderate	5 (0.8%)
Citalopram < = > Solifenacin	severe	severe	moderate	5 (0.8%)
Methylprednisolone < = > Tizanidine	severe	—	—	5 (0.8%)
Acetylsalicylic acid < = > Duloxetine	severe	moderate	mild	4 (0.6%)
Candesartan < = > Enoxaparin	severe	moderate	mild	4 (0.6%)
Citalopram < = > Fampridine	severe	—	—	4 (0.6%)
Diclofenac < = > Enoxaparin	moderate	severe	moderate	4 (0.6%)
Diclofenac < = > Methylprednisolone	severe	moderate	—	4 (0.6%)
Escitalopram < = > Pantoprazole	severe	—	mild	4 (0.6%)
Ramipril < = > Teriflunomide	—	severe	—	4 (0.6%)
Ramipril < = > Tizanidine	moderate	severe	mild	4 (0.6%)
Acetylsalicylic acid < = > Teriflunomide	—	severe	mild	3 (0.5%)
Acetylsalicylic acid < = > Venlafaxine	severe	moderate	mild	3 (0.5%)
Amlodipine < = > Magnesium	severe	—	—	3 (0.5%)
Baclofen < = > Levodopa	severe	moderate	moderate	3 (0.5%)
Bisoprolol < = > Tamsulosin	severe	—	mild	3 (0.5%)
Ciprofloxacin< = > Methylprednisolone	severe	severe	mild	3 (0.5%)
Citalopram < = > Dronabinol	severe	moderate	mild	3 (0.5%)
Citalopram < = > Mitoxantrone	severe	—	—	3 (0.5%)
Duloxetine < = > Ibuprofen	severe	moderate	mild	3 (0.5%)
Enoxaparin < = > Valsartan	severe	moderate	mild	3 (0.5%)
Escitalopram < = > Fingolimod	severe	severe	moderate	3 (0.5%)
Escitalopram < = > Ibuprofen	severe	moderate	mild	3 (0.5%)
Insulin glargine < = > Ramipril	severe	moderate	mild	3 (0.5%)
Methylprednisolone < = > Teriflunomide	—	severe	—	3 (0.5%)
Mitoxantrone < = > Solifenacin	severe	—	—	3 (0.5%)
Solifenacin < = > Torasemide	severe	—	—	3 (0.5%)

The medication schedules of a total of 627 patients with CIS/MS were evaluated using three drug interaction databases. This table lists 43 pDDIs (sorted by frequency) that were classified as severe in at least one of the databases and that were found for *n* ≥ 3 patients. Please note that the severity levels from Drugs.com (minor, moderate and major) and MediQ (low, average and high) were relabeled here according to those from Stockley’s. Disease-modifying drugs for MS are marked in bold. — = not recorded in the database; CIS, clinically isolated syndrome; MS, multiple sclerosis; pDDI, potential drug-drug interaction.

**TABLE 3:** Potential drug-drug interactions detected in only one database and classified as severe.

Severe pDDIs according to Stockley’s only	Frequency, *n* (%)	Severe pDDIs according to Drugs.com only	Frequency, *n* (%)
Methylprednisolone < = >Solifenacin	9 (1.4%)	Ibuprofen < = > Teriflunomide	6 (1.0%)
Mitoxantrone < = > Ondansetron	7 (1.1%)	Ramipril < = > Teriflunomide	4 (0.6%)
Methylprednisolone < = > Tizanidine	5 (0.8%)	Methylprednisolone < = > Teriflunomide	3 (0.5%)
Citalopram < = > Fampridine	4 (0.6%)	Candesartan < = > Potassium	2 (0.3%)
Amlodipine < = > Magnesium	3 (0.5%)	Cannabidiol < = > Teriflunomide	2 (0.3%)
Citalopram < = > Mitoxantrone	3 (0.5%)	Fingolimod < = > Methylprednisolone	2 (0.3%)
Mitoxantrone < = > Solifenacin	3 (0.5%)	Acetaminophen/paracetamol < = > Leflunomide	1 (0.2%)
Solifenacin < = > Torasemide	3 (0.5%)	Acetaminophen/paracetamol < = > Teriflunomide	1 (0.2%)
Dipyrone/metamizole < = > Prednisolone	2 (0.3%)	Acetylsalicylic acid < = > Brinzolamide	1 (0.2%)
Escitalopram < = > Fampridine	2 (0.3%)	Acetylsalicylic acid < = > Dorzolamide	1 (0.2%)
Mitoxantrone < = > Tolterodine	2 (0.3%)	Budesonide < = > Natalizumab	1 (0.2%)
Mitoxantrone < = > Torasemide	2 (0.3%)	Captopril < = > Teriflunomide	1 (0.2%)
Sodium < = > Torasemide	2 (0.3%)	Cladribine < = > Fluticasone	1 (0.2%)
Timolol < = > Travoprost	2 (0.3%)	Codeine < = > Tizanidine	1 (0.2%)
Beclometasone < = > Escitalopram	1 (0.2%)	Diclofenac < = > Teriflunomide	1 (0.2%)
Betamethasone < = > Dipyrone/metamizole	1 (0.2%)	Dimenhydrinate < = > Potassium citrate	1 (0.2%)
Betamethasone < = > Fenoterol	1 (0.2%)	Fingolimod < = > Tamoxifen	1 (0.2%)
Betamethasone < = > Fluconazole	1 (0.2%)	Ibuprofen < = > Immunoglobulin G	1 (0.2%)
Betamethasone < = > Formoterol	1 (0.2%)	Irbesartan < = > Potassium	1 (0.2%)
Bicalutamide < = > Goserelin	1 (0.2%)	Mirabegron < = > Tamoxifen	1 (0.2%)
Bicalutamide < = > Triptorelin	1 (0.2%)	Potassium < = > Solifenacin	1 (0.2%)
Budesonide < = > Venlafaxine	1 (0.2%)	Quetiapine < = > Tapentadol	1 (0.2%)
Caffeine < = > Paroxetine	1 (0.2%)	Topiramate < = > Trospium chloride	1 (0.2%)
Candesartan < = > Ramipril	1 (0.2%)		
Citalopram < = > Fludrocortisone	1 (0.2%)		
Citalopram < = > Hydrocortisone	1 (0.2%)		
Citalopram < = > Xipamide	1 (0.2%)		
Dexamethasone < = > Opipramol	1 (0.2%)		
Dydrogesterone < = > Topiramate	1 (0.2%)		
Eprosartan < = > Tamsulosin	1 (0.2%)		
Escitalopram < = > Methylprednisolone	1 (0.2%)		
Etofenamate < = > Fluoxetine	1 (0.2%)		
Etoricoxib < = > Methylprednisolone	1 (0.2%)		
Fenoterol < = > Fluconazole	1 (0.2%)		
Fingolimod < = > Sulpiride	1 (0.2%)		
Fingolimod < = > Tolterodine	1 (0.2%)		
Fludrocortisone < = > Solifenacin	1 (0.2%)		
Furosemide < = > Levofloxacin	1 (0.2%)		
Hydrocortisone < = > Solifenacin	1 (0.2%)		
Hydrocortisone < = > Tolterodine	1 (0.2%)		
Latanoprost < = > Timolol	1 (0.2%)		
Lovastatin < = > Niacin	1 (0.2%)		
Methylprednisolone < = > Quinine sulfate	1 (0.2%)		
Mitoxantrone < = > Tizanidine	1 (0.2%)		
Prednisolone < = > Solifenacin	1 (0.2%)		
Simvastatin < = > Sitagliptin	1 (0.2%)		
Tolterodine < = > Torasemide	1 (0.2%)		

In the dataset of 627 patients, we found 47 severe pDDIs in the Stockley’s database that were not listed in the other two databases. Similarly, we found 23 severe pDDIs in the Drugs.com database that were not listed in the other two databases. Among the 473 pDDIs that were found exclusively in the MediQ database, there was no severe pDDI. Disease-modifying drugs for multiple sclerosis are marked in bold. pDDI, potential drug-drug interaction.

### Factors associated with the risk of having a severe potential drug-drug interactions

Over all patients, we identified an average of 5.7 ± 9.4 pDDIs (0.9 ± 2.0 severe pDDIs) that were reported in at least one of the three drug interaction databases. For 441 of the 627 patients (70.3%), we found at least one pDDI, and for 221 patients (35.2%), we found at least one severe pDDI. The latter number is essentially the result of using Drugs.com and Stockley’s as only a small subset of 11 patients were found to have a severe pDDI according to MediQ.

**FIGURE 4:**
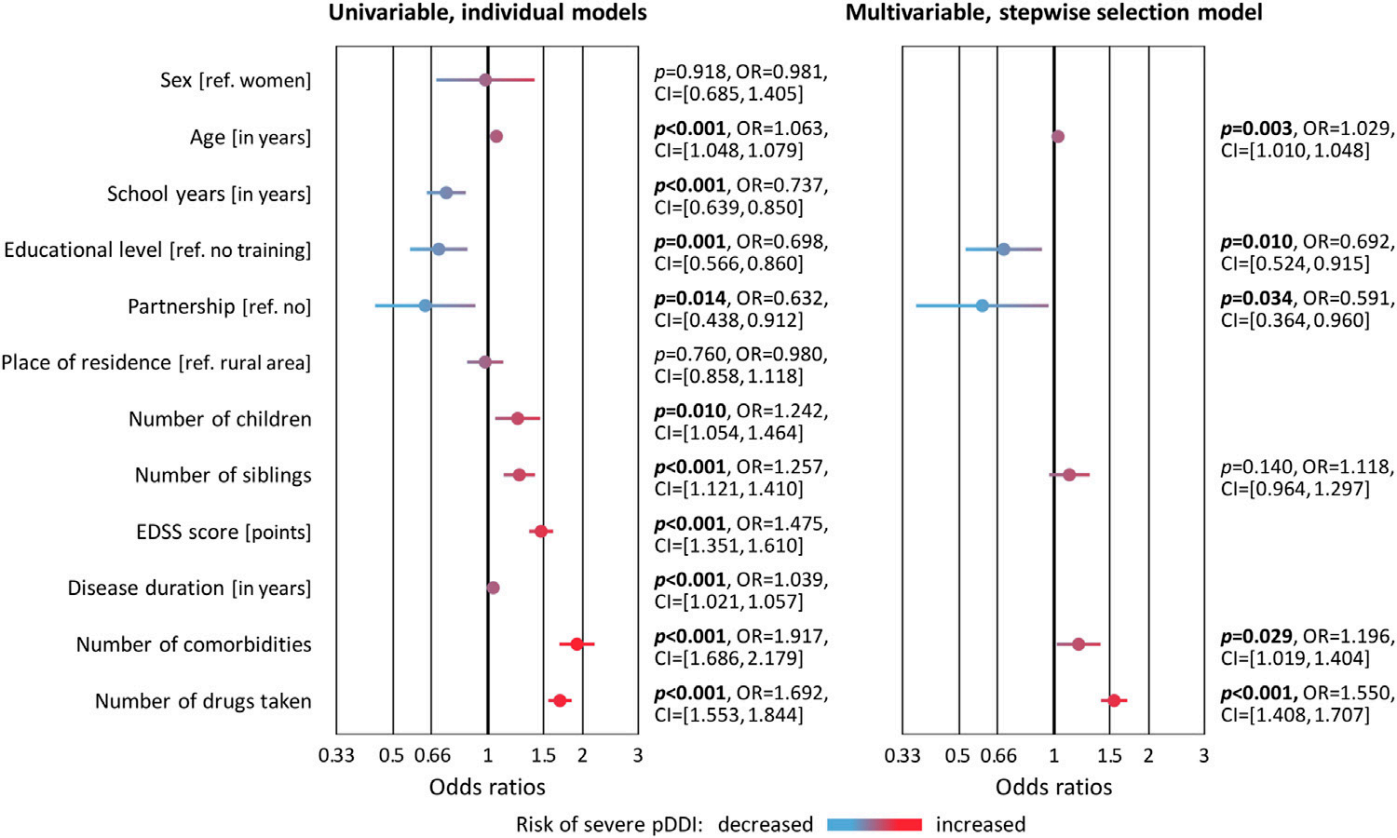
Factors associated with the occurrence of severe potential drug-drug interactions in patients with multiple sclerosis. A subset of 221 of the 627 patients had at least one severe pDDI according to at least one of the three databases used. Binary logistic regression analyses were performed to test for 12 variables whether they are associated with the risk of having a severe pDDI. This was done using each variable individually (univariable models) and the best predictive subset of variables (stepwise selection model). The ORs and 95% CIs from these models are shown as forest plots. *p*-values <0.05 are marked in bold. CI, confidence interval; EDSS, Expanded Disability Status Scale; OR, odds ratio; pDDI, potential drug-drug interaction; ref., reference.

**FIGURE 5:**
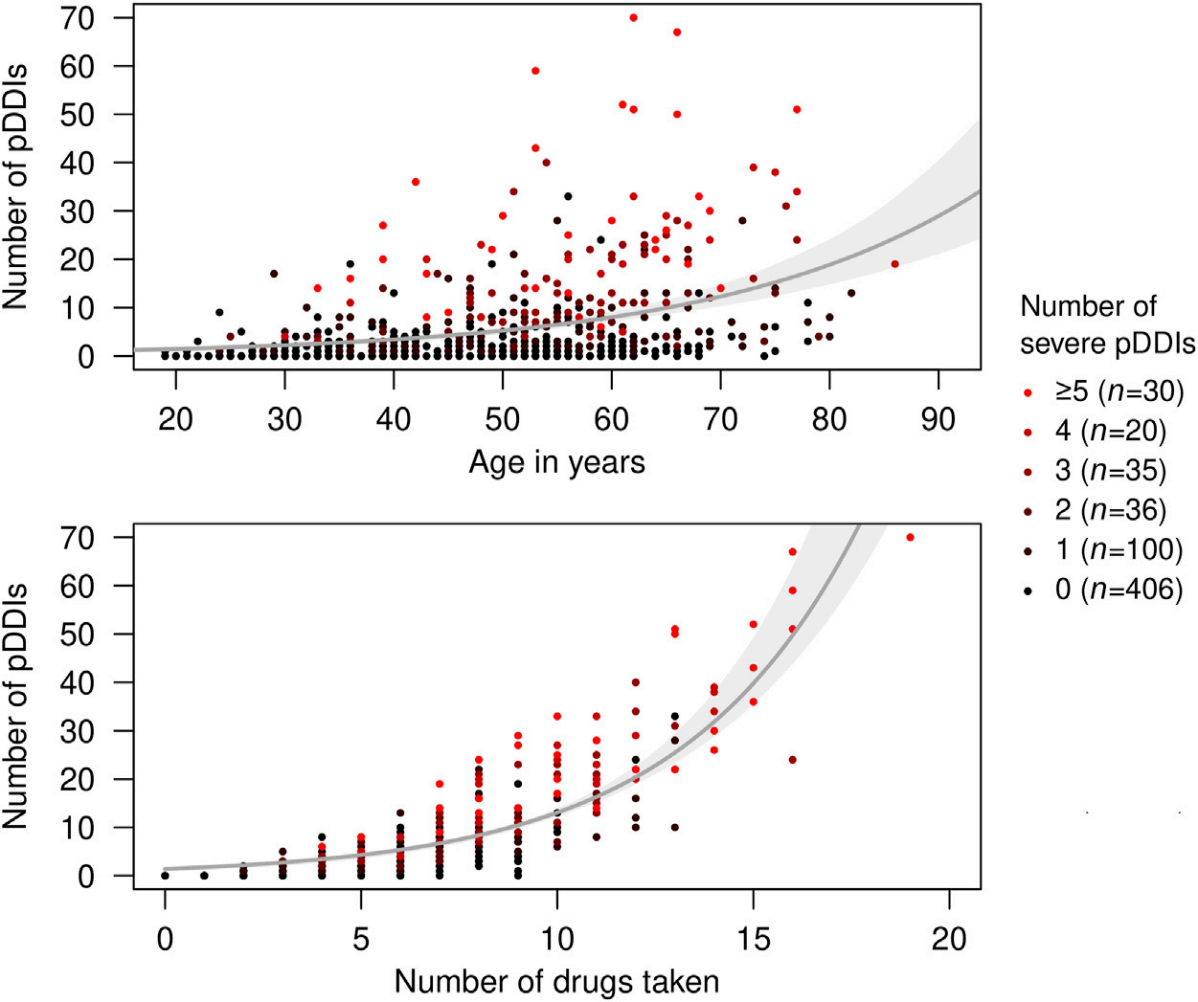
Frequency of potential drug-drug interactions in relation to age and number of drugs taken. The patients (*N* = 627) took 5.3 medications on average. A subset of 441 patients had at least one pDDI according to at least one of the three databases used. Red dots in the scatterplot represent patients with multiple severe pDDIs. Fitted exponential curves with 95% bootstrap confidence intervals are shown in gray. pDDI, potential drug-drug interaction.

The logistic regression analyses revealed predictors of the risk of having at least one severe pDDI. In the univariable models, statistically significant ORs >1 were obtained for age and disease duration, number of children and siblings, degree of disability (EDSS score), comorbidities as well as the number of drugs taken. Conversely, more years in school, a higher educational level and living in a partnership turned out to be protective factors with significant ORs < 1 (Figure 4). In the multivariable model, age, educational level, partnership status, comorbidities and number of drugs taken remained as significantly associated with the risk of having a severe pDDI. Multicollinearity was not detected in the data (VIF< 1.81). The particularly strong relationships between age and number of drugs taken with pDDI count are shown in Figure 5. Remarkably, one female SPMS patient taking 19 drugs had as many as 70 pDDIs, 22 of which were severe pDDIs. Another woman with SPMS received only four drugs (citalopram, mitoxantrone, ondansetron and solifenacin) but nonetheless had six severe pDDIs (all possible pairwise drug combinations) according to Stockley’s.

## Discussion

Patients with MS are typically treated with a broad spectrum of medications. In addition to DMDs, symptomatic drugs and CAMs are often used to alleviate the symptoms of MS, while comorbidities need to be treated with medications as well. This poses a significant risk of pDDIs, which can lead to adverse health outcomes. Therefore, as part of the therapy management, it should be regularly checked whether pDDIs are present, e.g., using pDDI screening tools. To provide insights into their utility, we here compared the databases Stockley’s, Drugs.com and MediQ with respect to differences in the detection and rating of pDDIs in patients with MS. We found that the databases provide quite heterogeneous information and that each database reports pDDIs that are not recorded in the other two databases. Beyond this database comparison, we discuss below the most frequent severe pDDIs identified in our patients and highlight sociodemographic and clinical characteristics that were associated with the occurrence of severe pDDIs.

With an average age of 48.6 years, a sex ratio of approximately 2.4 (female) to 1 (male) and a proportion of patients with relapsing-onset MS of ∼90%, our study cohort compares well with large national MS cohorts (Boström et al., 2013; Weih et al., 2020; Ohle et al., 2021). We thus believe that we can to some extent generalize the results of our study to a wider population of patients with MS. After combining the information from the three databases, the analysis revealed a prevalence of 70.3% and 35.2% of having ≥ 1 pDDI and ≥ 1 severe pDDI, respectively. However, only 18.9% of all different pDDIs were detected with all three pDDI screening tools used. It has been previously shown that there are large variations between CDSS/DDIDs concerning severity ratings and the documentation of information related to clinical effects, mechanism and management of pDDIs (Wang et al., 2010; Monteith and Glenn, 2019; Shariff et al., 2021). In fact, in studies comparing different pDDI programs and databases, the overlap of pDDIs that were detected in all resources ranged between 5% and 44% (Vonbach et al., 2008; Smithburger et al., 2010; Smithburger et al., 2012; Amkreutz et al., 2017; Fung et al., 2017; Sancar et al., 2019; Suriyapakorn et al., 2019; Tecen-Yucel et al., 2020; Prely et al., 2022). We found the lowest concordance rate for Drugs.com vs. MediQ (23.3%), and only 3.3% of the different pDDIs were recorded and classified with the same severity in all three databases. This finding is similar to earlier studies by Smithburger et al. (2010) who reported that the interaction databases Micromedex and Lexi-Interact agreed on the severity ratings in only ∼20% of the pDDIs and that some major pDDIs occurring in intensive care units were identified in only one of the two databases (Smithburger et al., 2012). In another study comparing Micromedex, Medscape and Drugs.com in the community pharmacy setting, 13.1% of all different pDDIs were scored with the same severity level in all three programs (Sancar et al., 2019).

There are multiple reasons for the limited overlap and concordance between the three databases considered in our study. First of all, there is no standardized definition of a pDDI (Hines et al., 2012), which leads to different views on what might be a pDDI and what not. Different databases may be based on different sources of information and set different requirements for the level of evidence to define a pDDI for a drug combination. Case reports may be sufficient for one database, while other databases may rather rely on pharmacokinetic properties (e.g., knowledge of CYP isozymes involved in the metabolism of the drugs) or studies on pharmacodynamic responses. Whether a drug interacts with another often depends on various factors (e.g., drug intake interval, dose and route of administration), which are not uniformly taken into account in the databases. With regard to the severity rating of pDDIs, there is also no consistent definition of, e.g., a mild pDDI. Another possible explanation for the diverging results is the different target group of each resource. MediQ is targeted at medical professionals and is intended for everyday clinical use, whereas Drugs.com is mainly build for patients and non-medical people. Drugs.com might therefore be more restrained in showing pDDIs than MediQ, because medical laypersons are usually less interested in any mild pDDI that might occur under certain circumstances and they would be otherwise confused by the amount of information (Weingart et al., 2003; Kusch et al., 2018). For patients, it is more important that they will be informed on possibly severe pDDIs so that they visit their doctor once more rather than not often enough, even if the likelihood of a pDDI to be actually life-threatening is low (Hammar et al., 2021). It also has to be considered that the databases are not equally complete regarding drugs and pDDIs recorded. For instance, in Drugs.com, dimetindene or fenoterol were not found and pDDIs could therefore not be determined for those. The update intervals differ and, therefore, a given pDDI might be documented differently across the databases. There is also typically more information on drugs that have been approved for a longer time (such as interferon beta) than for newer drugs (such as cladribine). For clinicians, it is thus currently recommended to use more than one CDSS/DDID and to consult a clinical pharmacist in order not to miss relevant pDDIs (Smithburger et al., 2010; Wang et al., 2010; Kheshti et al., 2016; Sancar et al., 2019; Suriyapakorn et al., 2019).

Severe pDDIs can lead to life-threatening conditions and require medical intervention to prevent serious consequences (Sheikh-Taha and Asmar, 2021). In our study, a total of 336 pDDIs were classified as severe in at least one database. The most common severe pDDI was acetylsalicylic acid with enoxaparin. This combination may lead to an increased bleeding tendency (Théroux et al., 1988). Citalopram, a selective serotonin reuptake inhibitor (SSRI), was most frequently involved in severe pDDIs. This finding is similar to a study on severe pDDIs in patients with dementia, according to which citalopram was involved in half of the top ten severe pDDIs (Bogetti-Salazar et al., 2016). We found severe pDDIs with citalopram for 33 different drugs, including mitoxantrone, fingolimod, acetylsalicylic acid, isoniazid and solifenacin. Citalopram is metabolized by the CYP2C19 enzyme, which is increased in activity after acetylsalicylic acid intake (Chen et al., 2003) but inhibited by isoniazid (Desta et al., 2001). Therefore, it may be appropriate to monitor the levels of citalopram in plasma or serum in the early phase of treatment. Dose adjustments may prevent later treatment failure and adverse drug reactions (Ostad Haji et al., 2013). The therapeutic reference range for citalopram is between 50 and 110 ng/ml, while concentrations > 220 ng/ml are considered to be above the “laboratory alert level” (Hiemke et al., 2018). Apart from pharmacokinetic interactions, SSRI medications are associated with a modest increase in the risk of gastrointestinal bleeding, and when used in combination with non-steroidal anti-inflammatory drugs (e.g., acetylsalicylic acid) or oral anticoagulants (e.g., phenprocoumon) the risk of bleeding complications is elevated (Anglin et al., 2014; Nochaiwong et al., 2022). Therefore, co-prescription should be weighed by a risk-benefit assessment. Solifenacin is used to relieve symptoms of an overactive bladder in patients with MS (van Rey and Heesakkers, 2011), but it may in rare cases cause a prolongation of the QT interval (Bray and Hancox, 2017). Citalopram also causes a dose-dependent QT interval prolongation (Maljuric et al., 2015). Hence, concurrent administration of citalopram and solifenacin can result in a higher risk of cardiac arrhythmias (Behr and Roden, 2013). Due to the relatively high prevalence of depressive and anxiety disorders in patients with MS (up to 50%), antidepressants such as SSRIs are often prescribed (Patten et al., 2017). To prevent severe pDDIs, individualized therapy with antidepressants should thus be implemented with critical indication and consideration of alternatives (Stamoula et al., 2021).

We found several severe pDDIs involving DMDs, e.g., teriflunomide, fingolimod, mitoxantrone and interferon beta. For the corticosteroid methylprednisolone, we found severe pDDIs with fingolimod and teriflunomide in Drugs.com. Fingolimod reversibly reduces the number of circulating lymphocytes, while teriflunomide reduces the proliferation of activated B and T lymphocytes (Bar-Or and Li, 2021). Studies found no generally increased risk of infections in patients treated with fingolimod or teriflunomide (Francis et al., 2014; Winkelmann et al., 2016; Winkelmann et al., 2022). However, concurrent use of immunomodulatory or immunosuppressive therapies can have additive effects on the immune system, thereby increasing infectious risks. Therefore, corticosteroid treatment for relapses should be limited (3–5 days) in MS patients receiving DMDs, and a decision for prolonged or repeated high-dose corticosteroid use should be made on an individual basis after careful consideration (Arvin et al., 2015; Abrantes et al., 2021). The pDDI resulting from the combination of citalopram with fingolimod was classified as severe due to the risk of ventricular arrhythmias, but clinical studies revealed no additional risk of abnormal electrocardiogram findings in patients who received fingolimod and SSRIs compared with patients receiving fingolimod therapy alone (Bermel et al., 2015; Bayas et al., 2016).

Older age and a higher number of comorbidities were strong risk factors for the occurrence of severe pDDIs according to the multivariable model. Furthermore, we found severe pDDIs more frequently in MS patients with a lower educational level and in patients who were not in a partnership. This is in line with previous studies by our group and others showing that with older age and the presence of comorbidities, the number of drugs taken increases on average (Frahm et al., 2019; Frahm et al., 2020b; Zanghì et al., 2021; Bachmann et al., 2022) and so does the risk of pDDIs (Debus et al., 2022). Our analysis also complements the results of studies not related to MS. An Irish study of elderly community dwellers found that patients with a higher educational level were less likely to have severe pDDIs (Hughes et al., 2021). In patients with dementia, factors that were associated with severe pDDIs were taking a greater number of drugs, depression, dementia severity and caregiver burden (Bogetti-Salazar et al., 2016).

To prevent adverse drug reactions due to (severe) pDDIs, the treating physicians should regularly review the current medication plan and educate the patient well about the correct use of drugs (e.g., dosage and intake interval) and side effects that may occur (Tannenbaum and Sheehan, 2014). In this effort, the physicians should not only pay attention to the medications they prescribed, but should also place these in a critical context with the medications prescribed by physicians from other specialties. When checking for pDDIs, the use of OTC drugs should not be neglected (Scherf-Clavel, 2022) as, according to our previous study, about one in five pDDIs is related to OTC medicines in patients with MS (Bachmann et al., 2022). If a clinically relevant pDDI is identified, there are various options for dealing with it. Rx and OTC medications that are not necessary for the patient can be discontinued. Depending on the need, the use of a drug can also be reduced or just temporarily suspended. Substitution of a drug with an alternative, less interacting drug might also be conceivable. If all this is not possible after weighing the risks, a close therapy monitoring supported by laboratory tests and a detailed counseling of the patient should be ensured. It is particularly important that the patient knows the typical first signs of adverse events associated with an unavoidable pDDI so that a physician consultation is sought quickly if the need arises. A close cooperation between different medical disciplines and between physicians and pharmacists should be understood as the basis for improving individualized patient care.

Our study has several limitations. First, the data were collected at medical centers in Germany, but internationally, there are differences in the therapeutic management of patients with MS and in the provision and reimbursement of drugs. When collecting the medication data, it was ensured that the data were recorded twice (*via* the patient interview and the patient record). Nevertheless, there is always a risk of inaccuracies when analyzing medication schedules. For the evaluation of pDDIs, we here gathered and compared information from three selected commonly used databases. The discussed severe pDDIs therefore do not necessarily represent an exhaustive list of all severe pDDIs that may occur in MS patients. In the present study, we did not investigate possible drug-food and drug-gene interactions. Moreover, we did not examine whether the treating physicians were already aware of the pDDIs and whether they considered them as not clinically relevant. Some of the identified pDDIs are based on theoretical mechanisms involving known CYP enzyme substrates, inducers or inhibitors, but are currently without solid evidence to affirm the theoretical interaction (by clinically relevant case reports). We cannot state to what extent the differences in the detection of pDDIs between the databases were due to insufficient data on the pharmacokinetics or pharmacodynamics of the drugs. The mechanisms of action of individual pDDIs were reported quite differently in the databases. In some cases only pharmacokinetic mechanisms were explained, in others only pharmacodynamic mechanisms. We also did not record actual adverse drug events in the patients, which is an issue that would be ideally pursued further in a longitudinal study. Thus, additional studies are warranted to examine how pDDI resources can be better integrated in routine clinical practice to provide a quick overview on unwanted effects and serious problems related to inappropriate drug use in MS patients. In the future, patient safety might be improved by machine learning methods, which can help in predicting relevant interactions between multiple drugs (Basile et al., 2019; Han et al., 2022). Further research might also involve the patients and investigate whether they are aware of the problem and understand information about pDDIs (Hammar et al., 2021).

In conclusion, our study provides a comprehensive comparison of the three pDDI screening tools Stockley’s, Drugs.com and MediQ based on a sample of 627 patients. A total of 1,684 different pDDIs were identified, with large differences between the databases in the number of pDDIs recorded (range: 706–1,161). Due to the heterogeneity in the classification of pDDI severities, only six of the 336 different severe pDDIs were rated as such in all three databases. In our patient cohort, citalopram was the drug most frequently involved in different severe pDDIs. Overall, 35.2% of the 627 patients had at least one severe pDDI, the occurrence of which was significantly associated with older age, lower educational level, living without a partner, comorbidities and the number of medications taken. In the context of chronic diseases such as MS, polypharmacy and the assessment of pDDIs present major challenges that could be better addressed through improved digital health solutions. When searching for pDDIs, it is currently recommended to check more than one database to increase sensitivity. Periodic medication reviews by the treating physicians and appropriate reductions or substitutions of medications can reduce the risk of severe pDDIs and improve the therapy management.

## Data Availability

The raw data supporting the conclusion of this article will be made available by the authors, without undue reservation.

## Supplementary material

SUPPLEMENTAL FIGURE S1 Network visualization of severe potential drug-drug interactions detected in patients with multiple sclerosis. A total of 1,684 different pDDIs were recorded for the 627 patients in this study. This graph shows a subset of 336 pDDIs that were classified as severe in at least one of the three databases used. The interactions (edges) connect 164 different active drug ingredients (nodes), including 7 DMDs for MS. The size of the nodes corresponds to the number of different pDDIs in which the drug is involved. The thickness of the edges indicates the frequency of the pDDIs in the patient cohort. The color of the edges indicates the consistency of the severity rating across the databases. DMD, disease-modifying drug; MS, multiple sclerosis; pDDI, potential drug-drug interaction.

## Abbreviations

AIC, Akaike information criterion; CAM, complementary and alternative medicines; CDSS, clinical decision support software; CI, confidence interval; CIS, clinically isolated syndrome; CYP, cytochrome P450 superfamily; DDID, drug-drug interaction database; DMD, disease-modifying drug; EDSS, Expanded Disability Status Scale; MS, multiple sclerosis; *n*/*N*, number; OR, odds ratio; OTC, over-the-counter; pDDI, potential drug-drug interaction; PPMS, primary progressive multiple sclerosis; ref, reference; RRMS, relapsing-remitting multiple sclerosis; Rx, prescription drug; SPMS, secondary progressive multiple sclerosis; SSRI, selective serotonin reuptake inhibitor; VIF, variance inflation factor; vs, versus.
