# Supplementary figures and images for: Screening for severe drug-drug interactions in patients with multiple sclerosis: A comparison of three drug interaction databases
Source: Front Pharmacol. 2022 Aug 5;13:946351. doi: 10.3389/fphar.2022.946351 (PMC9416235; doi:10.3389/fphar.2022.946351)

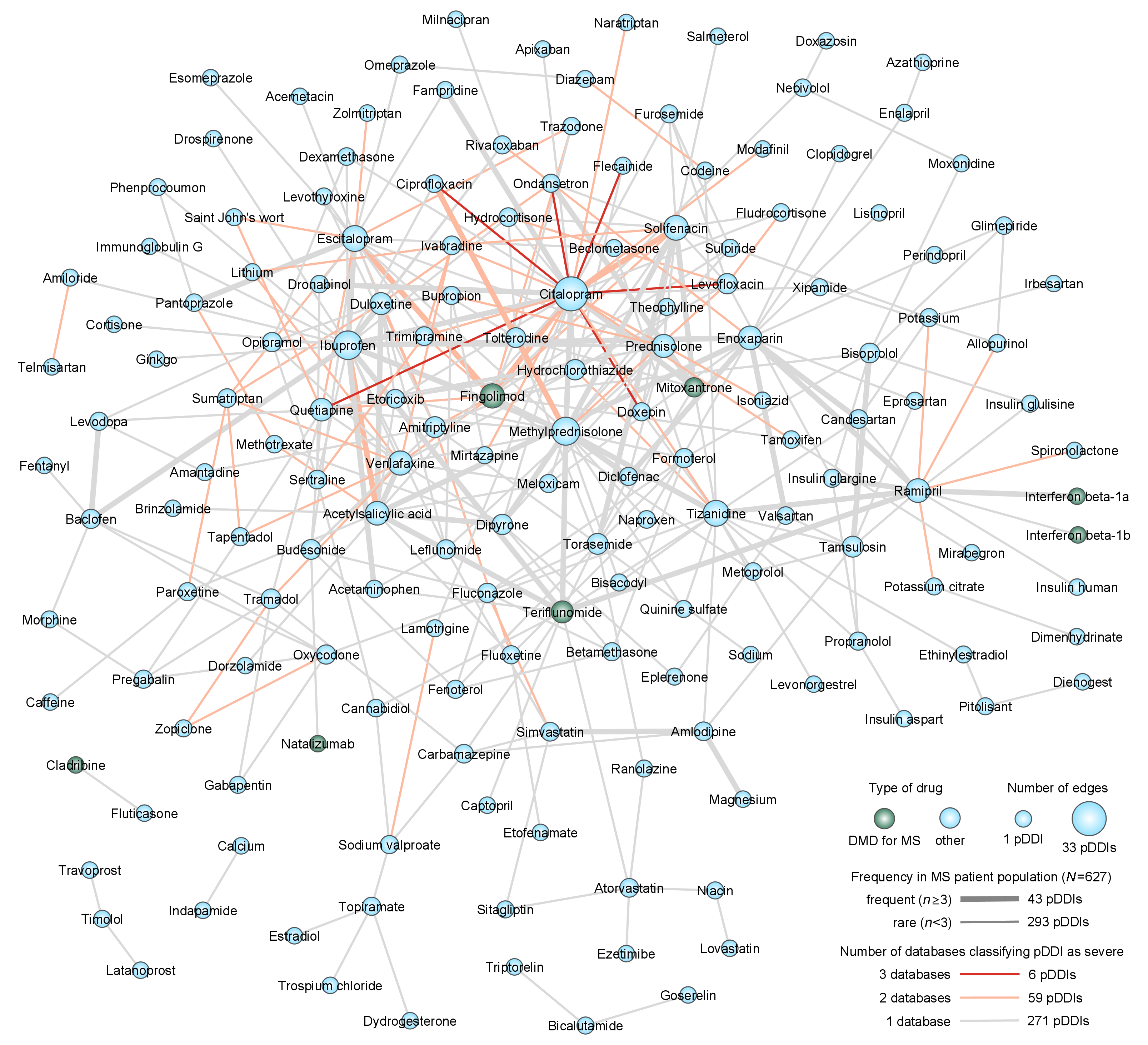

Supplement: Supplementary file 1 [file Image1.TIF]
